# The Relationship Between Insomnia and Cognitive Impairment in Breast Cancer Survivors

**DOI:** 10.1093/jncics/pkz041

**Published:** 2019-06-07

**Authors:** Kevin T Liou, Tim A Ahles, Sheila N Garland, Q Susan Li, Ting Bao, Yuelin Li, James C Root, Jun J Mao

**Affiliations:** 1 Integrative Medicine Service; 2 Department of Psychiatry and Behavioral Sciences; 3 Breast Medicine Service; 4 Department of Epidemiology and Biostatistics; 5 Memorial Sloan Kettering Cancer Center, New York, NY; 6 Department of Psychology, Memorial University of Newfoundland, St. John’s, Newfoundland, Canada

## Abstract

**Background:**

Cancer-related cognitive impairment is an emerging public health burden. Growing research suggests that sleep disturbances contribute to poor cognition. Our study aimed to evaluate the association between insomnia and cognitive impairment in breast cancer survivors.

**Methods:**

We analyzed cross-sectional data from a cohort study of postmenopausal women with stage 0–III hormone receptor-positive breast cancer on aromatase inhibitor therapy. The study was conducted between November 2011 and April 2015 at an academic cancer center (Philadelphia, PA). Insomnia was assessed with the Insomnia Severity Index. Perceived cognitive impairment was assessed with the cognitive subscale of the Breast Cancer Prevention Trial Symptom Checklist. We used linear regression to evaluate the association between insomnia and perceived cognitive impairment.

**Results:**

Among 1072 patients, 556 (51.9%) reported insomnia and 847 (79.0%) were bothered by cognitive symptoms (forgetfulness, difficulty concentrating, distractibility). Greater perceived cognitive impairment was reported by patients with mild insomnia (regression coefficient [β] = 0.35, 95% confidence interval [CI] = 0.23 to 0.46, *P *<* *.001), moderate insomnia (β = 0.51, 95% CI = 0.36 to 0.65, *P *<* *.001), and severe insomnia (β = 0.94, 95% CI = 0.67 to 1.21, *P *<* *.001), compared with those without insomnia. Greater perceived cognitive impairment was also associated with patients younger than 55 years (β = 0.30, 95% CI = 0.15 to 0.45, *P *<* *.001), taxane-based chemotherapy (β = 0.11, 95% CI = 0.004 to 0.22, *P *=* *.04), anxiety (β = 0.47, 95% CI = 0.30 to 0.64, *P *<* *.001), and depression (β = 0.65, 95% CI = 0.35 to 0.94, *P *<* *.001).

**Conclusions:**

Among postmenopausal breast cancer survivors receiving aromatase inhibitor therapy, insomnia and cognitive impairment are prevalent and characterized by a graded association, in which severity of perceived cognitive impairment increases as insomnia severity increases. Our findings warrant further research to determine whether addressing sleep is a strategy to improve management of cancer-related cognitive impairment.

Characterized by problems with memory, concentration, executive function, and/or psychomotor skills (1,2), cognitive impairment affects approximately 50% of breast cancer survivors (3), resulting in poorer quality of life, impaired daily functioning, and delayed recovery (4–6). The development of cognitive symptoms may contribute to early discontinuation of aromatase inhibitor (AI) therapy (7,8), thereby increasing risk of breast cancer recurrence and mortality (9). This issue is particularly relevant for postmenopausal women with hormone receptor-positive tumors, who comprise the majority of breast cancer survivors, and for whom standard of care is AI therapy for up to 10 years (10). Despite growing research, only a few pharmacologic (11) and nonpharmacologic (12) interventions have demonstrated promising outcomes for cancer-related cognitive impairment. Identifying clinical factors associated with cognitive impairment in breast cancer survivors may help facilitate the development of new management strategies for this debilitating condition.

Insomnia affects up to 60% of the cancer population (13), with breast cancer patients reporting the highest number of sleep-related complaints (14). One of the most common daytime correlates of insomnia is impaired cognitive ability (15). Substantial research suggests that sleep disturbances contribute to poor cognitive function (16–18). In a meta-analysis of 24 studies conducted in noncancer populations, those with insomnia were found to have cognitive impairment of small to moderate magnitude compared with normal sleepers (19). Few studies have specifically examined the relationship between cognitive impairment and insomnia in the breast cancer population. In a United Kingdom study of 142 women with breast cancer who received adjuvant chemotherapy, self-reported sleep problems were statistically significantly associated with perceived concentration problems at 12 months after chemotherapy completion (20). Similarly, in another study of 67 French-Canadian women treated for breast cancer, those with insomnia demonstrated greater perceived cognitive impairment and worse objective performance on verbal episodic memory and executive functioning compared with those without insomnia (21). These results differ from those of an American study that evaluated 389 adults with diverse cancer types and found that sleep disturbances were not statistically significantly associated with performance on cognitive screening instruments (22).

These findings warrant further investigation to confirm the relationship between sleep and cognitive function in the breast cancer population. A robust association in an oncology setting would provide a compelling rationale to investigate whether addressing sleep disturbances can potentially improve management of cancer-related cognitive impairment, a prevalent, debilitating condition with limited treatment options. This study aimed to evaluate the magnitude of association between self-reported insomnia and perceived cognitive impairment in a large cohort of breast cancer survivors receiving adjuvant AI therapy.

## Methods

### Study Design and Population

We analyzed cross-sectional data from the Wellness After Breast Cancer study, an ongoing cohort study of postmenopausal breast cancer survivors receiving AI therapy. The purpose of the Wellness After Breast Cancer study is to evaluate risk factors associated with overall symptom burden and how these symptoms affect clinical outcomes related to breast cancer. Patients were recruited from the Abramson Cancer Center (ACC) of the University of Pennsylvania (Philadelphia, PA) between November 2011 and April 2015 with follow-up ongoing. Patients were eligible if they were English speaking; were postmenopausal women with a history of histologically confirmed stage 0 to III, hormone receptor-positive breast cancer; were taking a third-generation AI (anastrozole, letrozole, or exemestane); and had completed chemotherapy, radiotherapy, and/or surgery at least 1 month prior to study enrollment. Women with metastatic breast cancer (stage IV) were excluded from the study. Research staff obtained permission from the primary oncologist, met with patients at their oncology appointments, obtained written informed consent, and distributed a self-administered survey. For those who could not complete the survey in the clinic, a stamped envelope with a return address was provided for them to mail back the survey. The institutional review board of the University of Pennsylvania and the ACC Clinical Trials Scientific Review and Monitoring Committee approved the study.

### Perceived Cognitive Impairment (Primary Outcome)

The primary outcome was perceived cognitive impairment as measured by the cognitive subscale on the Breast Cancer Prevention Trial (BCPT) Symptom Checklist. This three-item subscale asks patients to rate on a five-point Likert scale (0 = not at all, 1 = slightly, 2 = moderately, 3 = quite a bit, 4 = extremely) how bothered they were by three cognitive symptoms (forgetfulness, difficulty concentrating, and easily distracted) during the past 4 weeks. A total mean score was calculated from the scores of each cognitive symptom, with higher scores indicating a greater degree of perceived cognitive impairment. The BCPT has been validated and used in breast cancer populations to assess cognitive symptoms (23,24). The Cronbach α for the cognitive subscale is 0.85, indicating good reliability (24).

### Insomnia (Primary Exposure)

The primary exposure of interest was self-reported insomnia as measured by the Insomnia Severity Index (ISI). The ISI is a seven-item instrument that has been demonstrated to be a reliable and valid measure of insomnia in cancer populations with a Cronbach α of 0.90 (25). The widely accepted ISI score cutoffs are 0–7 (no sleep difficulties), 8–14 (mild insomnia), 15–21 (moderate insomnia), and 22–28 (severe insomnia) (25–27).

### Covariates

Participants self-reported sociodemographic variables (age, race and ethnicity, and education level) and specific clinical factors (body mass index and smoking and/or alcohol status). Cancer-related variables (tumor stage, years since diagnosis, previous and/or current cancer treatments, and AI type and duration of use) were assessed by medical chart abstraction. To evaluate for presence of mood symptoms, we used the Hospital Anxiety and Depression Scale, which is a 14-item, self-administered rating scale with two subscales (anxiety and depression), each containing seven items. The reliability, validity, and factor structure of the Hospital Anxiety and Depression Scale has been established in cancer patients (28,29). Scores of 11 or greater on either subscale are considered to be clinically abnormal psychological morbidity, whereas scores of 8–10 indicate borderline cases and 0–7 are considered normal.

### Statistical Analysis

Descriptive statistics were calculated as means and percentages. Bivariate analyses were conducted to examine whether insomnia severity and other variables were associated with the primary outcome. Variables associated with the primary outcome at *P* less than .10 in the bivariate analyses were included in multivariable linear regression analyses to estimate the regression coefficients of variables that were independently associated with the primary outcome. All analyses were two-sided. *P* values less than .05 indicated statistical significance. We compared the *R^2^* values of three different multivariable linear regression models to estimate the variance in perceived cognitive impairment that could be explained by insomnia and the other variables. Model 1 included insomnia as the only variable. Model 2 added sociodemographic and clinical variables to Model 1. Model 3 added anxiety and depression to the variables in Model 2; the results of Model 3 were summarized in tabular format. To assess for multicollinearity, we calculated variance inflation factors for the variables in Model 3 to ensure that they were within an acceptable range (ie, <5). All statistical analyses were conducted using STATA (Windows version 12.0, StataCorp LLC, College Station, TX).

## Results

### Patient Characteristics

**Figure 1.**
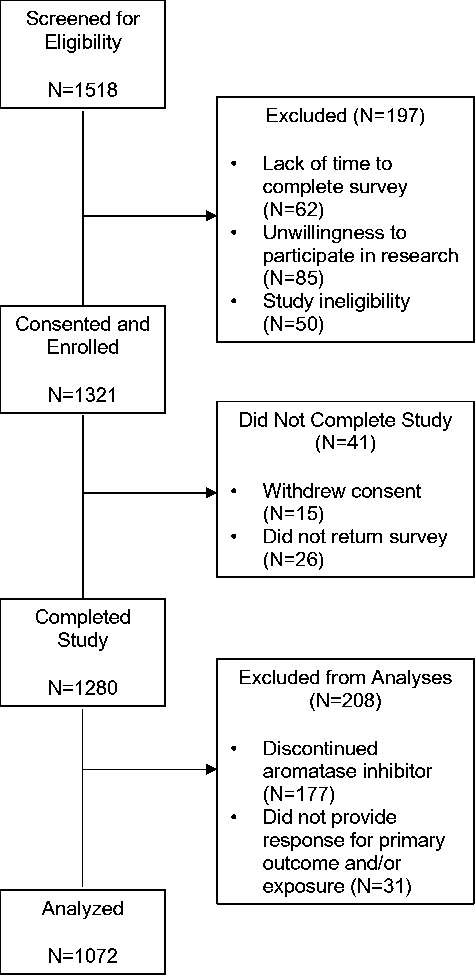
Screening, enrollment, and completion of study.

We screened a total of 1518 consecutive breast cancer survivors (Figure 1) . Of these, 1321 (87.0%) agreed to participate and provided consent, and 197 (13.0%) did not participate because of lack of time to complete the survey (n = 62), unwillingness to participate in research (n = 85), or study ineligibility (n = 50). After enrollment, 41 patients did not complete the study because of withdrawal of consent (n = 15) or failure to return the survey (n = 26), resulting in a total sample of 1280 patients with an overall completion rate of 96.8%. Of these 1280 patients, an additional 208 were excluded from analyses because they discontinued AI therapy (n = 177) or because they did not complete the BCPT and/or ISI questionnaires (n = 31), resulting in a final sample size of 1072.

Among 1072 breast cancer survivors, the mean age (SD) was 62.1 (9.9) years, 888 (82.8%) were white, and 557 (52.0%) had received chemotherapy. The other clinical characteristics of the study population are listed in Table 1.

**Table 1.** Patient characteristics by the three-item Breast Cancer Prevention Trial (BCPT) cognitive subscale scores*

Characteristic	No. (%)	Mean (SD) BCPT cognitive subscale score	*P*
Insomnia†			<.001
None (0–7)	516 (48.13)	0.65 (0.73)	
Mild (8–14)	343 (32.00)	1.15 (0.90)	
Moderate (15–21)	167 (15.58)	1.43 (0.84)	
Severe (22–28)	46 (4.29)	2.24 (1.14)	
Age, y			<.001
>65	363 (33.86)	0.80 (0.84)	
55–65	501 (46.74)	1.02 (0.91)	
<55	208 (19.40)	1.32 (0.97)	
Race			.58
White	888 (82.84)	0.99 (0.91)	
Non-white	184 (17.16)	1.04 (0.94)	
Education			.96
Did not graduate college	434 (40.52)	1.00 (0.94)	
Graduated college	637 (59.48)	1.00 (0.90)	
Body mass index, kg/m^2^			.24
<25	416 (38.81)	0.95 (0.89)	
25–30	315 (29.38)	1.00 (0.91)	
>30	341 (31.81)	1.06 (0.95)	
Smoking			.36
Never smoked	576 (53.83)	1.03 (0.93)	
Current or former smoker	494 (46.17)	0.97 (0.90)	
Alcohol			.50
<1 drink daily	885 (82.63)	1.01 (0.92)	
≥1 drink daily	186 (17.37)	0.96 (0.90)	
Cancer stage			.42
0 and I	551 (51.98)	1.00 (0.95)	
II	370 (34.91)	0.96 (0.85)	
III	139 (13.11)	1.08 (0.94)	
Years since cancer diagnoses			.97
<2	473 (44.12)	1.01 (0.90)	
2–5 y	415 (38.71)	1.00 (0.95)	
>5	184 (17.16)	0.99 (0.89)	
Chemotherapy			.002
None	515 (48.04)	0.91 (0.90)	
Chemotherapy without taxane	103 (9.61)	0.99 (0.83)	
Taxane-based chemotherapy	454 (42.35)	1.11 (0.94)	
Radiotherapy			.10
None	304 (28.36)	1.07 (0.97)	
Yes	768 (71.64)	0.97 (0.89)	
Surgery			.002
Lumpectomy	620 (57.89)	0.93 (0.89)	
Mastectomy	451 (42.11)	1.10 (0.94)	
Current aromatase inhibitors (AI)			1.00
Anastrozole	863 (80.88)	1.00 (0.94)	
Exemestane	57 (5.34)	1.00 (0.78)	
Letrozole	147 (13.78)	1.00 (0.82)	
Years of AI use			.27
>3	269 (25.09)	0.94 (0.89)	
1–3	546 (50.93)	1.00 (0.93)	
<1	257 (23.97)	1.07 (0.91)	
Anxiety			<.001
No (HADS <8)	731 (68.77)	0.78 (0.80)	
Borderline (8–10)	206 (19.38)	1.31 (0.96)	
Clinically abnormal (≥11)	126 (11.85)	1.79 (0.91)	
Depression			<.001
No (HADS <8)	933 (88.02)	0.89 (0.83)	
Borderline (8–10)	92 (8.68)	1.73 (0.94)	
Clinically Abnormal (≥11)	35 (3.30)	2.22 (1.22)	

* Variables associated with the primary outcome at *P* < .10 were included in the multivariable linear regression analyses (Table 2). AI = aromatase inhibitor; HADS = Hospital Anxiety and Depression Scale.

† Insomnia Severity Index.

### The Relationship Between Insomnia and Perceived Cognitive Impairment

In our study population, the mean BCPT cognitive score was 1.00 (0.92), which is equivalent to being “slightly” bothered by cognitive difficulties, based on the five-point scale (0 = not at all, 1 = slightly, 2 = moderately, 3 = quite a bit, 4 = extremely). In total, 847 (79.0%) reported that they were bothered by a cognitive problem (ie, score ≥1 on at least one of the BCPT cognitive items); specifically, 798 (74.4%) were bothered by forgetfulness, 626 (57.5%) were bothered by concentration issues, and 598 (55.8%) were bothered by distractibility. Of the 847 reporting cognitive difficulties, 390 (46.0%) reported that they were at least “moderately” bothered by their symptoms (ie, score ≥2 on at least one of the BCPT cognitive items). Regarding sleep difficulties, 556 (51.9%) reported insomnia (ie, score ≥8 on ISI): 343 (32.0%) had mild insomnia, 167 (15.6%) had moderate insomnia, and 46 (4.3%) had severe insomnia.

**Figure 2.**
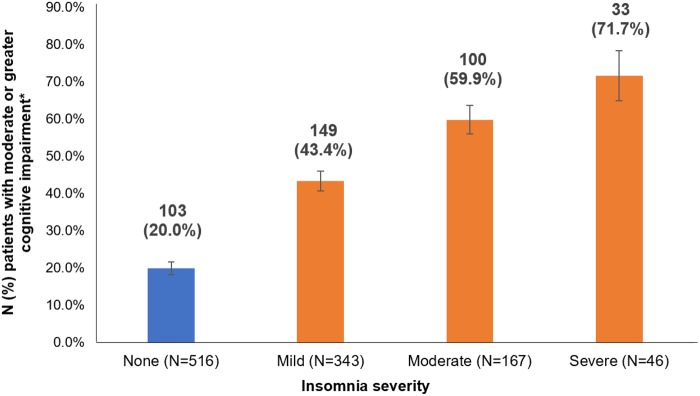
Prevalence of moderate or greater perceived cognitive impairment among breast cancer survivors by severity of comorbid insomnia. *Score ≥2 on at least one of the Breast Cancer Prevention Trial cognitive items (ie, forgetfulness, concentration, distractibility) indicates moderate or greater perceived cognitive impairment.

**Figure 3.**
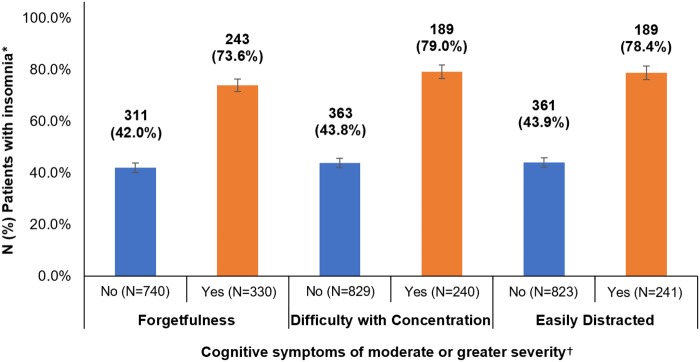
Prevalence of insomnia among breast cancer survivors with moderate or greater perceived cognitive impairment. *Insomnia is defined as score ≥8 on the Insomnia Severity Index. †Score ≥2 on a Breast Cancer Prevention Trial cognitive item (ie, forgetfulness, concentration, distractibility) indicates moderate or greater severity of the cognitive symptom. Because of missing data, some variables do not add up to 1072.

In bivariate analyses (Table 1), higher ISI scores were associated with higher mean BCPT cognitive scores, indicating that patients with greater severity of insomnia symptoms were more likely to report a greater degree of perceived cognitive impairment. For example, 71.7% of patients with severe insomnia reported that they were at least “moderately” bothered by a cognitive symptom, compared with only 20.0% of patients without insomnia (Figure 2). Similarly, insomnia was more prevalent among patients who were at least “moderately” bothered by cognitive symptoms compared with those who were not bothered or only slightly bothered by cognitive symptoms (ie, score <2 on all BCPT cognitive items) (Figure 3).

When adjusted for all covariates (Table 2), the association between ISI scores and BCPT cognitive scores remained statistically significant. The mean BCPT cognitive scores increased as insomnia severity increased from mild (regression coefficient [β] = 0.35, 95% confidence interval [CI] = 0.23 to 0.46; *P *<* *.001) to moderate (β = 0.51, 95% 0.36 to 0.65; *P *<* *.001) to severe (β = 0.94, 95% CI = 0.67 to 1.21; *P *<* *.001). Based on these findings, patients with severe insomnia were more likely to report a BCPT cognitive score that was approximately one point higher on the five-point scale compared with those without insomnia.

**Table 2.** Linear regression analyses of patient characteristics associated with the three-item Breast Cancer Prevention Trial (BCPT) cognitive subscale scores

Characteristic	Unadjusted β coefficient (95% CI)	*P*	Adjusted β coefficient (95% CI)	*P*
Insomnia†				
None (0–7) (reference)	—	—	—	—
Mild (8–14)	0.49 (0.38 to 0.61)	<.001	0.35 (0.23 to 0.46)	<.001
Moderate (15–21)	0.78 (0.64 to 0.92)	<.001	0.51 (0.36 to 0.65)	<.001
Severe (≥22)	1.58 (1.33 to 1.83)	<.001	0.94 (0.67 to 1.21)	<.001
Age, y				
>65 (reference)	—	—	—	—
55–65	0.22 (0.10 to 0.34)	<.001	0.15 (0.04 to 0.26)	.01
<55	0.52 (0.37 to 0.68)	<.001	0.30 (0.15 to 0.45)	<.001
Chemotherapy				
None (reference)	—	—	—	—
Chemotherapy without taxane	0.09 (–0.10 to 0.28)	.37	0.10 (−0.16 to 0.18)	.91
Taxane-based chemotherapy	0.20 (0.09 to 0.32)	.001	0.11 (0.004 to 0.22)	.04
Radiotherapy				
None (reference)	—	—	—	—
Yes	−0.1 (−0.22 to 0.02)	.10	–0.03 (–0.15 to 0.08)	.57
Surgery				
Lumpectomy (referent)	—	—	—	—
Mastectomy	0.18 (0.07 to 0.29)	.002	0.05 (–0.06 to 0.17)	.35
Anxiety				
No (HADS <8) (referent)	—	—	—	—
Borderline (8–10)	0.52 (0.39 to 0.65)	<.001	0.27 (0.14 to 0.40)	<.001
Clinically abnormal (≥11)	1 (0.84 to 1.16)	<.001	0.47 (0.30 to 0.64)	<.001
Depression				
No (HADS <8) (referent)	—	—	—	—
Borderline (8–10)	0.84 (0.66 to 1.03)	<.001	0.41 (0.23 to 0.60)	<.001
Clinically abnormal (≥11)	1.33 (1.04 to 1.62)	<.001	0.65 (0.35 to 0.94)	<.001

* This table summarizes the results of the linear regression analyses described as Model 3 in the manuscript. The variance inflation factors for the variables ranged from 1.15 to 1.51, indicating that multicollinearity was not a problematic issue in this regression model. Empty cells are marked with “—” to indicate reference group. CI = confidence interval; HADS = Hospital Anxiety and Depression Scale.

† Insomnia Severity Index.

### Other Factors Associated With Perceived Cognitive Impairment

In addition to insomnia, several other sociodemographic and clinical factors were associated with the primary outcome (Table 2). After adjusting for all covariates, greater perceived cognitive impairment was reported by patients younger than 55 years (β = 0.30, 95% CI = 0.15 to 0.45; *P *<* *.001) and those between ages 55 and 65 years (β = 0.15, 95% CI = 0.04 to 0.26; *P *=* *.01) compared with those older than 65 years. Perceived cognitive impairment did not differ by race or education level. Compared with those who had not undergone treatment with chemotherapy, patients treated with taxane-based chemotherapy reported greater perceived cognitive impairment (β = 0.11, 95% CI = 0.004 to 0.22; *P *=* *.04). There were no statistically significant differences between other treatment-related variables, such as AI type, duration of AI treatment, or prior history of radiation or surgery. Greater perceived cognitive impairment was reported by patients with borderline (β = 0.27, 95% CI = 0.14 to 0.40; *P *<* *.001) or clinically abnormal (β = 0.47, 95% CI = 0.30 to 0.64; *P *<* *.001) anxiety and those with borderline (β = 0.41, 95% CI = 0.23 to 0.60; *P *<* *.001) or clinically abnormal (β = 0.65, 95% CI = 0.35 to 0.94; *P *<* *.001) depression compared with those without any mood symptoms.

#### Variance in Perceived Cognitive Impairment.

With insomnia as the only variable, Model 1 explained 19% of the variance in perceived cognitive impairment (*R^2^* = 0.19). When sociodemographic and clinical variables were added to insomnia, Model 2 explained an additional 3% of the variance (*R^2^* = 0.22). After adding depression and anxiety to the variables in Model 2, Model 3 explained an additional 6%, accounting for a total of 28% of the variance in perceived cognitive impairment (*R^2^* = 0.28).

## Discussion

In this cross-sectional analysis of more than 1000 postmenopausal breast cancer survivors receiving AI therapy, both insomnia and perceived cognitive impairment were prevalent and characterized by a graded association that remained statistically significant after adjusting for all covariates. Perceived cognitive impairment was also associated with younger age, taxane-based chemotherapy, anxiety, and depression; however, the magnitudes of these associations were smaller in comparison with that of the association between perceived cognitive impairment and insomnia. These findings contribute to our current understanding of cancer-related cognitive impairment as a multifactorial condition (1,30,31) and describe an important relationship between sleep and cognition in breast cancer survivors that warrants further research.

The prevalence of insomnia and cognitive impairment in our study population was comparable to other epidemiologic studies. We found that more than 50% reported insomnia and nearly 80% of patients were bothered by perceived cognitive impairment. In previous studies of breast cancer patients, the prevalence of insomnia was estimated to range from 19% to 61% (32,33), and the rates of self-reported cognitive decline ranged from 37% to 71% (3,20). The variation in observed prevalence rates may reflect the heterogeneity of the breast cancer study population as well as the differences in instruments used to assess insomnia and cognitive impairment. These findings support the clinical importance of screening both for sleep and cognitive issues in breast cancer survivors.

Consistent with two earlier studies (20,21), we identified a robust association between insomnia and perceived cognitive impairment in breast cancer survivors. These findings are in line with prior research of noncancer populations that demonstrated a statistically significant link between sleep disturbances and poor cognitive function (16–19). Our study provides further evidence that the relationship between sleep and cognition is characterized by a graded association in breast cancer survivors, with the severity of perceived cognitive impairment increasing as the severity of self-reported insomnia increased. This echoes another study conducted in patients without cancer, in which chronic sleep deprivation resulted in cumulative, dose-dependent deficits in cognitive performance (34).

Although the exact mechanisms have not been fully defined, growing research has identified several potential biological pathways by which sleep disturbances may contribute to cognitive dysfunction, including increased amyloid-β deposition (35), alterations in neurotransmitter systems (eg, γ-aminobutyric acid, cyclic adenosine monophosphate, brain-derived neurotrophic factor) (36–38), dysregulation of hypothalamic-pituitary-adrenal axis (39), and neuro-inflammation and disrupted neurogenesis in the hippocampus (40–42). An association between insomnia and reduced hippocampal volumes has been demonstrated in some (43–45) but not all (46) brain imaging studies of noncancer patients. Other studies have shown that sleep deprivation affects areas of the brain involved in working memory (47–49). Taken together, this emerging evidence provides biological plausibility that addressing sleep disturbances can potentially improve cancer-related cognitive impairment, but further research is needed in oncology settings, because processes related to cancer and its treatment may affect the pathways linking sleep and cognition.

A recent systematic review identified preliminary evidence that cognitive-behavioral therapy for insomnia (CBT-I) produces small to moderate beneficial effects on self-reported cognitive functioning, including among cancer patients (50). In one study of 56 breast cancer survivors with insomnia, CBT-I produced a trend toward statistically significant greater improvement in the Attentional Function Index score compared with behavioral placebo control (51). Another study of women with nonmetastatic breast cancer and comorbid insomnia demonstrated a significant improvement in the cognitive subscale of the European Organisation for Research and Treatment of Cancer Quality-of-Life Questionnaire – Core 30 (EORTC QLQ-C30) after receiving CBT-I (52). These findings need to be confirmed in adequately powered randomized controlled trials to determine whether management of cancer-related cognitive impairment can be improved by targeting sleep issues; future research should also compare CBT-I with other interventions that have proven efficacy for insomnia.

Although sleep disturbances have traditionally been viewed as mere manifestations and secondary features of mood disorders (53), recent research has shown that insomnia has a unique etiology and pathophysiology distinct from other psychiatric conditions (54–56). Consistent with this more recent conceptualization of insomnia, our study demonstrated a robust association between insomnia and perceived cognitive impairment that remained statistically significant after adjusting for anxiety and depression. Our findings contribute to the well-established literature linking mood and cognition (57–59), including among breast cancer patients (60), and suggests that sleep also warrants attention in future research on cancer-related cognitive impairment.

Our findings should be considered in the context of several limitations. First, our study did not incorporate any objective neurocognitive testing and instead relied on self-report, which may be subject to recall bias. However, Savard and Ganz (61) have compellingly argued that subjective measures are more clinically useful than objective measures, particularly with regards to evaluating the impact of cognitive impairment on physical functioning and quality of life. Drawing parallels with sleep and depression research, they further stated that the subjective appraisal of cognitive abilities should be the primary indicator of cognitive impairment in epidemiological studies and clinical trials and can be reliably measured with validated instruments. Indeed, perceived cognitive impairment has been shown in some studies to be a better predictor of structural brain damage or cognitive decline compared with objective performance on neurocognitive testing (62,63). Although we used a validated instrument to assess perceived cognitive impairment, the outcome measure contains only three items and thus may not fully capture all relevant cognitive domains. Future studies should aim to characterize the relationship between insomnia and cognitive impairment using more comprehensive, validated instruments that include subjective as well as objective measures of sleep and cognition.

Another limitation of our study is the cross-sectional design, which prevents us from elucidating any causal or temporal relationships between cognitive impairment and other factors. Additionally, our approach to constructing the regression models may have led to the exclusion of some clinically relevant variables in the analyses; thus, the possibility of residual confounding must be considered. Finally, this study was conducted at an academic center and included only postmenopausal breast cancer survivors receiving AI therapy, the majority of whom were white and well educated. Therefore, our results may not be generalizable to all breast cancer survivors, particularly those who are not receiving AI therapy. Given that an association between AI usage and cognitive impairment has been demonstrated in some (64,65) but not all (66–68) studies of patients with breast cancer, further research is needed to clarify whether AI therapy may contribute to cognitive impairment via estrogen depletion (69) or other biological mechanisms.

Despite these limitations, this large survey study of more than 1000 postmenopausal breast cancer survivors on AI therapy identified a statistically significant graded association between self-reported insomnia and perceived cognitive impairment after adjusting for covariates. Future studies should investigate the temporal relationship between these two symptoms as well as the causal mechanisms underlying the association between sleep and cognition in oncology settings. Research on interventions should also evaluate whether addressing sleep disturbances can potentially improve management of cancer-related cognitive impairment, a common but challenging condition to treat in breast cancer survivors.

## Funding

This work was supported by the National Institutes of Health (R01-CA158243 to JJM, P30-CA016520 to the Abramson Cancer Center, and P30-CA008748 to Memorial Sloan Kettering); the Laurance S. Rockefeller Fund (Memorial Sloan Kettering); and the Translational and Integrative Medicine Research Fund (Memorial Sloan Kettering).

## Notes

Affiliations of authors: Integrative Medicine Service (KTL, QSL, TB, JJM) and Department of Psychiatry and Behavioral Sciences (TAA, YL, JCR) and Breast Medicine Service (TB) and Department of Epidemiology and Biostatistics (YL), Memorial Sloan Kettering Cancer Center, New York, NY; Department of Psychology, Memorial University of Newfoundland, St. John’s, Newfoundland, Canada (SNG).

The authors declare no potential conflicts of interest with respect to the research, authorship, and/or publication of this article.

The funding sources were not involved in the study design; collection, analysis and interpretation of data; writing of the report; or decision to submit the article for publication.

The authors would like to thank the patients, oncologists, nurses, and clinical staff at the ACC of the University of Pennsylvania for their contributions to this study.
